# Application of Machine-Learning Methods to Recognize mitoBK Channels from Different Cell Types Based on the Experimental Patch-Clamp Results

**DOI:** 10.3390/ijms22020840

**Published:** 2021-01-15

**Authors:** Monika Richter-Laskowska, Paulina Trybek, Piotr Bednarczyk, Agata Wawrzkiewicz-Jałowiecka

**Affiliations:** 1 Institute of Physics, University of Silesia in Katowice, 40-007 Katowice, Poland; 2 Faculty of Science and Technology, University of Silesia in Katowice, 41-500 Chorzow, Poland; paulina.trybek@us.edu.pl; 3 Department of Physics and Biophysics, Institute of Biology, Warsaw University of Life Sciences—SGGW, 02-787 Warszawa, Poland; piotr_bednarczyk@sggw.pl; 4 Department of Physical Chemistry and Technology of Polymers, Silesian University of Technology, 44-100 Gliwice, Poland

**Keywords:** K-nearest neighbors algorithm, autoencoder, machine learning, mitoBK channels, gating dynamics

## Abstract

(1) Background: In this work, we focus on the activity of large-conductance voltage- and Ca${}^{2+}$-activated potassium channels (BK) from the inner mitochondrial membrane (mitoBK). The characteristic electrophysiological features of the mitoBK channels are relatively high single-channel conductance (ca. 300 pS) and types of activating and deactivating stimuli. Nevertheless, depending on the isoformal composition of mitoBK channels in a given membrane patch and the type of auxiliary regulatory subunits (which can be co-assembled to the mitoBK channel protein) the characteristics of conformational dynamics of the channel protein can be altered. Consequently, the individual features of experimental series describing single-channel activity obtained by patch-clamp method can also vary. (2) Methods: Artificial intelligence approaches (deep learning) were used to classify the patch-clamp outputs of mitoBK activity from different cell types. (3) Results: Application of the K-nearest neighbors algorithm (KNN) and the autoencoder neural network allowed to perform the classification of the electrophysiological signals with a very good accuracy, which indicates that the conformational dynamics of the analyzed mitoBK channels from different cell types significantly differs. (4) Conclusion: We displayed the utility of machine-learning methodology in the research of ion channel gating, even in cases when the behavior of very similar microbiosystems is analyzed. A short excerpt from the patch-clamp recording can serve as a “fingerprint” used to recognize the mitoBK gating dynamics in the patches of membrane from different cell types.

## 1. Introduction

Artificial intelligence (AI) approaches (machine learning, neural networks) are still gaining popularity in biological sciences due to their utility in diagnosing, managing, and designing drugs against many popular diseases [1,2,3,4,5]. Ion channels play an important role in many physiological processes and are considered to be drug targets [6]. Therefore, the use of AI methods in investigation of these transport proteins seems very promising.

In this work we investigate the activity of large-conductance voltage- and Ca${}^{2+}$-activated potassium channels (BK) from the inner mitochondrial membrane (mitoBK) [7,8,9]. Initially, the BK channels were investigated in plasma-membrane [10,11,12]. In that research the name of the channel-BK was derived from its big single-channel conductance (even 300 pS). In this work we consider the mitochondrial variants of the BK channels (mitoBK). The mitoBK channels play a crucial role in the mitochondrial potassium influx, and consequently, regulate the membrane potential or the matrix volume [7,9,13]. It was also shown that activation of the mitoBK channels fosters cytoprotection [14]. Moreover, many reports in the literature postulate the occurrence of structural and functional coupling of the mitoBK channel with the mitochondrial respiratory chain and involvement of these channels in generation of reactive oxygen species [15,16].

The mitoBK channels are encoded by the same gene as their cell membrane counter-parts-BK, i.e., the Kcnma1 (Slo1) gene [9]. Nevertheless, their exonic composition can vary in different cell types as a result of the alternative splicing during the Kcnma1 gene transcription [17,18], or post-translational modifications [19,20,21]. According to the literature, the mitoBK channels are expressed when the Kcnma1 undergoes splicing to the DEC isoform [22].

The channel proteins can be associated with different types of regulatory β (1–4) and γ (1–4) subunits [9,23,24,25,26]. Auxiliary subunits are exhibited in a tissue-specific manner. Thus, they are meaningful in defining the function of BK and mitoBK channels in different cell types [23].

Summing up, the variations in exonic composition as well as the differences in the leading type of α-β or α-γ complex result in a tissue-specific functional heterogeneity within the BK and mitoBK channels. Depending on the splice variant of the mitoBK channel or its association with auxiliary subunits gating kinetics (which describes the spontaneous changes of the conformational states of the channel observed as the conducting/non-conducting fluctuations) as well as the dependence of channel conductance on voltage ($U_{m}$) and Ca${}^{2+}$ concentration may significantly differ at quantitative level.

In this work our methodology aims at discriminating between gating dynamics of the mitoBK channels from different cell lines based on the inherent features of input data (a short excerpt from the raw patch-clamp recording). We expect the tissue-specific patterns of mitoBK current fluctuations. The different characteristics of time series of single-channel currents should be associated with a highly complex pattern of switching between possible protein conformations at the given conditions. In turn, the underlying conformational dynamics can be modulated, among others, by the interactions between the α and auxiliary subunits, lipid–protein or protein–protein interactions [27,28]. Thus, in the case of our paper, we show that a sole way of how the single channel fluctuates can be considered to be a “fingerprint” allowing identification of a type of cell from where the investigated channel stems.

The development of the machine-learning techniques to determine patterns within the experimental signal describing ion channel activation and gating with or without the use of a potential specific channel modulators (like e.g., natural flavonoids, toxins) may be useful in unraveling the molecular mechanisms of their interaction with a channel protein and choosing the most effective and specific activating/inhibiting substances acting on a particular BK channel variant. First, however one should provide a solid theoretical background for further investigations on the potential modulators of the ion channels. In that aim, presently the methods of artificial intelligence are used to recognize the general genetic, structural or functional features of ion channels [29,30]. In the work of Han et al. [29] the authors used the machine-learning techniques to predict the set of genes encoding different ion channel types. In turn, in the article of Celik et al. [30] the deep neural networks were used to detect single-molecule events (discernible states of the channel gate) from raw experimental data in the form of a time series of single-channel currents obtained by the patch-clamp method [31].

In this work, we aim to analyze by means of AI methods the single-channel activity patterns of the mitoBK channels from three different cell types: human endothelial cell line (EA.hy926), primary human dermal fibroblasts cell line (HDFa) and embryonic rat hippocampal neurons. The appropriate thorough electrophysiological analysis was carried out previously [32,33,34]. In every case, we adjusted the solutions used in experiment to ensure full calcium activation of the mitoBK channel (100 μM CaCl_2_ in case of endothelium and hippocampus; 200 μM CaCl_2_ for fibroblasts), and we compare the time series of single-channel currents recorded at 6 different membrane potential levels (i.e., −60, −40, −20, +20, +40 +60 [mV]) which actually cover a broad range of voltage-activation. Then, the patch-clamp signals of the mitoBK channels from different cell types were compared (at a training stage of machine learning). The separation of a test data after applying the AI ought to indicate the presence of any differences in gating dynamics of investigated groups of channels. Our former reports [32,33,34] suggest that the most striking difference between the analyzed groups of mitoBK channels is that they are co-assembled with different types of β subunits: β2 (in endothelium), β3 (in fibroblasts) or β4 (in hippocampus), which was confirmed by Western blot analysis [33], immunohistochemical labeling and other molecular biology methods, for details please see [32,33,34]. Thus, the recognized dynamical diversity may stem from other kinds of interactions within various mitoBK-β subunit complexes. Still, there can be also other factors that can exert some effects on the recognized dynamical features of the system, which are characteristic for different lines of wild-type cells. These are: different splice variants mixtures of BK channels in inner mitochondrial membranes from versatile types of cells and also differences in the lipid and protein composition of the investigated membranes. Nevertheless, this primary research is thought to be conducted using non-modified channel systems to show the applicability of the presented AI-based approach in analysis of real, complex microbiosystems.

To achieve our goals, the following strategy was undertaken:

1. the first data preprocessing stage-where the abnormal recordings were rejected from further analysis. This stage of analysis was performed by means of the autoencoder anomaly detection technique.
2. the second data preprocessing stage including partition of the recordings into smaller subseries (samples), dimensionality reduction by Piecewise Aggregate Approximation (PAA) and scaling
3. the cross-validation step: partition of the recordings into the training and test sets,
4. classification of tissue-specific single-channel activity patterns by means of the K-Nearest Neighbors (KNN) algorithm and evaluation of its accuracy,
5. visualization of the test data set compressed to a 2-dimensional space by means of the autoencoder,
6. calculation of the average distances between the signal samples corresponding to various mitoBK channel groups in the 2-dimensional latent space.

As one can see, our analysis consists of two parts. In the first part we involve the KNN algorithm to assign one of the three different categories (according to the cell types used in experimental part) for the preprocessed samples. In the second one, we compress the preprocessed samples with the autoencoder into a 2-dimensional space. Then we calculate the distance between the emerging clusters corresponding to the various mitoBK channel groups. The cluster centers are evaluated with the K-Means algorithm.

Our approach enables for a classification of raw experimental data without any initial assumptions about the system kinetics or its features. In this work, we presented a possible application of artificial neural network based on the unsupervised learning method, which allows us to analyze the data without knowing or defining a priori labels for the hidden structures within it. In this methodology, recognition of correlations between the analyzed data sets and finding features that actually correlate them, enable us to find some kind of patterns (some commonalities) within the analyzed signals.

One of the crucial stages of our analysis is an implementation of the widely used K-Nearest Neighbors (KNN) algorithm. In contrast to the standard linear methods it is more effective in grasping an internal structure of signal together with an insight into its complex nature. The effectiveness of this technique has been already confirmed by its great achievements in the field of electrophysiological time series classification [35,36,37]. Apart from the KNN method, we used an autoencoder neural network in two stages of our analysis. The first stage of initial data processing we pointed out the “outliers” and rejected of the abnormal recordings from the dataset, which was further processed. At the data preprocessing part, the autoencoder neural network was implemented to find outlier files. The main role of this algorithm is based on its possibility to compresses the data to a lower dimension, what was also used in further analysis. Specifically, we applied autoencoder in the next steps of investigation to reduce the dimensionality of our samples which allowed us to visualize them in a 2-dimensional space.

The autoencoder neural network is a deep learning technique that has been used to extract features and/or detect objects from different types of datasets. The possibilities of using this kind of network in the field of biological data analysis are large. It has been already applied in examination of different cancer types, electrophysiological signal classification, cell biology and gene structure investigation [38,39,40,41,42]. It performs well in the anomaly detection, identification of outliers in time series analysis [43]. In this work, we show its possible application in both identification of anomalies in the experimental set of patch-clamp recordings and in machine-learning methodology.

Most of the previous studies about the characteristics of BK channels dynamics do not take into account analysis of raw ion current sequences. From the biophysical point of view, the different characteristics of the time series of a single-channel current are associated with a highly complex pattern of switching between possible protein conformations. In this work, three different types of cells are classified by testing the dynamics of the same type of channel (mitoBK). This kind of classification could be the basis for the further use of machine learning in the various types of ion channel classification models, e.g., severity of disease, including the stage of cancer, e.g., glioblastoma cells, where the activity of BK channels is widely studied [9,15].

## 2. Results

### 2.1. Kinetic Properties of Experimental Data

**Figure 1:**
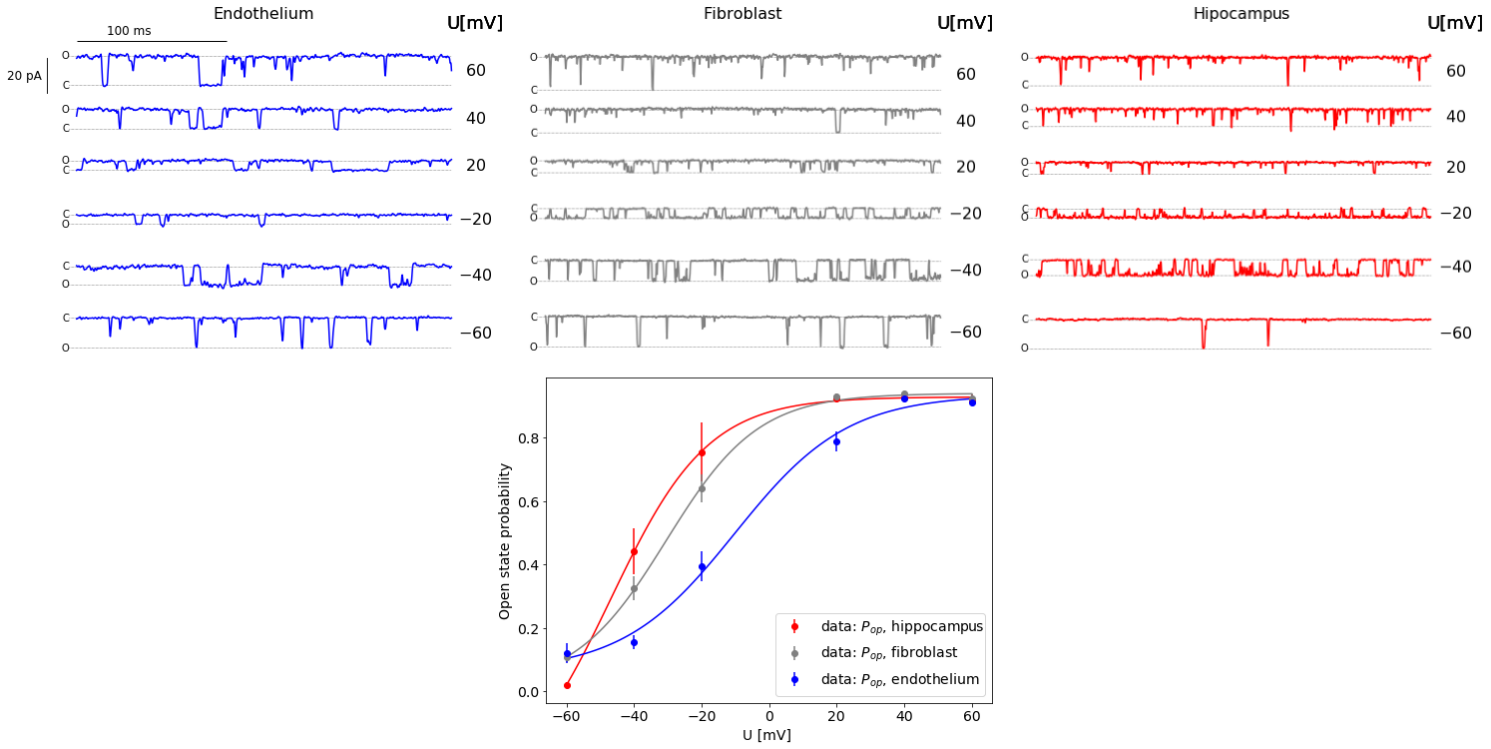
(**Upper**): the samples of the original ionic currents of single mitoBK channels for three different cell types: endothelium (blue); fibroblast (gray); hippocampus (red). (**Lower**): the average probability of channel opening at different membrane potentials fitted by sigmoidal curves.

To visualize the characteristics of the analyzed data, samples of experimental time series of single-channel currents and the corresponding voltage-activation curves are shown in Figure 1. As one can see, the examined mitoBK channels exhibit a typical conductance and highly fluctuating pattern of gating. There are only slight quantitative differences in sigmoidal dependencies of $p_{op}$ vs. $U_{m}$. The analyzed membrane potentials covered a broad range of voltage-activation of the channel.

Consistent activation curves were obtained and published in our previous papers, concerning activity of mitoBK channels [32,33,34]. Analyzing the relations between the $p_{op}$ vs. $U_{m}$ presented in aforementioned works, here we observe an analogous right-shift of activation curve corresponding to endothelial mitoBK channel in relation to the channels from other cell types. Also, the relative similarity of the $p_{op}\left(U_{m}\right)$ dependencies describing hippocampal and fibroblast patches are clearly visible.

### 2.2. Machine-Learning Results

In this subsection, we demonstrate the main results of this work. We classify, using KNN algorithm, samples obtained from the subseries of experimental recordings for three different cell lines at 6 levels of membrane potential. The results of this classification, i.e., prediction accuracies are presented in Table 1. For comparison, we made the calculations with and without the application of the anomaly detection technique at the initial processing stage. For details about the applied procedure, please refer to the Section 4.2.3.

We observe a significant improvement for the data preprocessed with the autoencoder (Table 1). In our opinion, it confirms the validity of the anomaly detection step (and rejection of the so-called outlier traces) during the data preparation.

The presence of the outlier traces can be explained by recording of single-channel currents describing some rarely expressed splice variants of the mitoBK channel, or catching the activity of a channel which undergoes some kind of protein–protein interactions. Nevertheless, this kind of behavior does not describe the majority of the mitoBK channel population from a given cell type. Thus, it is reasonable not to consider these outliers in further analysis. The rejection of anomalies allows us to classify the visibly homogeneous populations of tissue-specific variants of the mitoBK channels, which is well-founded from the biological point of view.

What is also worth noticing, is the increase in the prediction accuracy when the difference in electric potential across the membrane raises (i.e., at highly hyper- or depolarized membranes). At moderate membrane potential of −20 mV the accuracy with anomaly detection is the lowest (see Table 1). In these terms, the position of the voltage sensor can exert a relatively weak effect on the channel pore, so the energetic differences between channel conformations should be small. The potential barriers restricting the movement of the pore-gate domain ought to be sufficiently low to not hamper open–closed fluctuations of the gate. Thus, the switching dynamics between the channel conformations are so complex in all analyzed patches that some problems in correct classification may occur.

**Figure 2:**
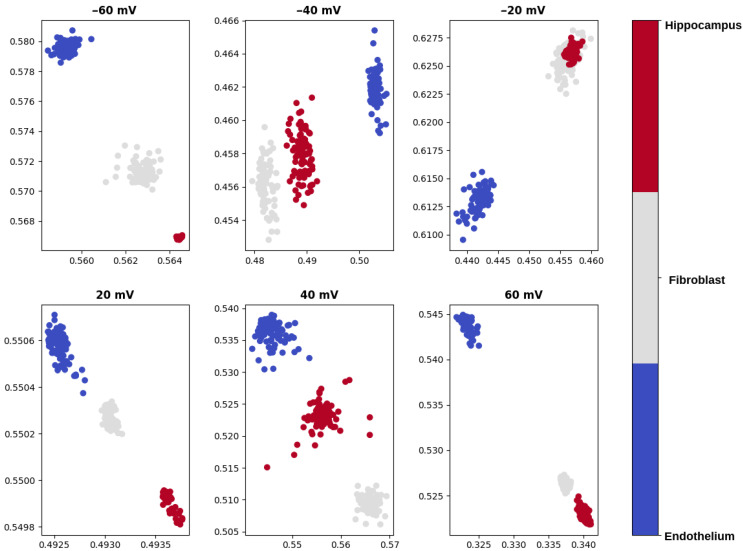
An exemplary visualization of the recordings reduced to a 2-dimensional space with the autoencoder corresponding to different pipette potentials and different cell types. Each dot represents a 1000-data-points-long subseries from the original recording. Some of the dots overlap each other.

**Figure 3:**
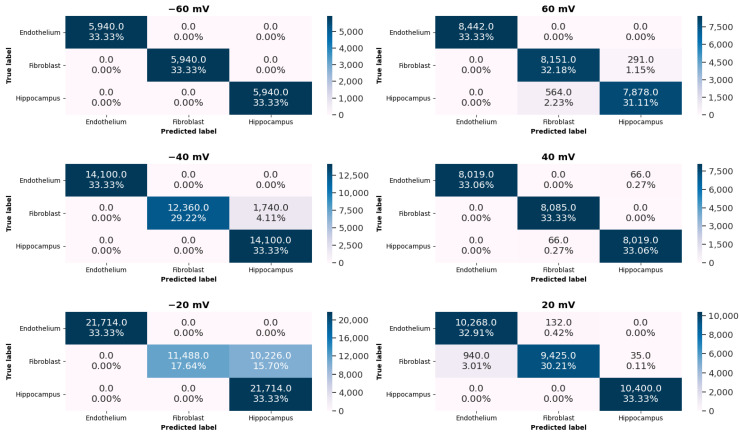
The set of confusion matrices for different pipette potentials. The values given in each block of the confusion matrices indicate the number and percentage of samples classified in a given category. Please note that the overall number of samples for each potential $U_{m}$ is different, but it is equally distributed among three cell lines.

As visualized in Figure 2 and Figure 3 and presented in Table 1, in general the clusters representing single-channel currents in investigated cell types are well separated.

To give some quantitative measure of distances between the clusters emerging in the 2-dimensional latent space (representing different cellular lines) we applied the K-Means clustering. Due to the fact that in our analysis there were 3 independent cell lines, we set the value of K=3. After finding the cluster centers we were able to calculate the average distances between centroids corresponding to the various cells. These results are presented in Table 1 and are denoted as Dist F, Dist FE, Dist HE characterizing distances between the appropriate pairs: fibroblast–hippocampus, fibroblast–endothelium and hippocampus–endothelium, respectively. The average distances in the latent space are the highest between the clusters representing hippocampal and endothelial cells for most membrane potentials (excluding $U_{m}$ = +40 mV). In turn, the clusters representing fibroblasts are located nearer the ones corresponding to the hippocampal cells than the ones corresponding to the endothelial cells for most values of the $U_{m}$ (The only exception is at $U_{m}$ = +20 mV, where the distances are comparable). These results indicate that the characteristics of channel activity pattern mostly differ from the other ones in case of the endothelial cells. Based on the obtained results, it is evident that some differences in the dynamics of the investigated groups of mitoBK channels can occur. These differences may be a manifestation of various mechanisms of switching between the channel’s stable conformations (which can be observed in the form of single-channel currents of discernible magnitude and duration).

Please note that during the analysis of the content of Table 1 the distances should not be compared directly between the different potentials. For each potential, an independent autoencoder net was applied for classification of the mitoBK channel activity patterns corresponding to the different cell lines.

To visualize the data separation, we additionally compress the test data measured at the same potential, but corresponding to the different cell types. The dimensionality reduction is performed by means of an autoencoder. Please note that the application of the autoencoder in our research is twofold. It is used both for the outlier detection and visualization of the samples corresponding to different cell types in a 2-dimensional space.

The analyzed data exhibit good separability in the introduced 2-dimensional space. However, according to our observations, the data are easily separable when they were not compressed. Thus, we used the reduced data only for the visualization purposes: the accuracy of KNN classification presented in Table 1 are calculated for the samples of dimension equal to 200, i.e., before the dimensionality reduction by the autoencoder neural network.

## 3. Discussion

Modern machine-learning techniques enable the classification, identification or interpretation of massive data sets. As a result, we are already witnessing the rapid adoption of machine-learning techniques as a useful research tool in the field of molecular biology and electrophysiology. This work follows this scientific trend. Analogously to the works of Han and Celik et al. [29,30], this research shows how ion channel electrophysiology can serve as a field of exploitation for the AI techniques. Specifically, the main aim of the current research was to apply artificial intelligence approaches to classify and discern from each other the experimental patch-clamp results describing mitoBK activity in different cell types. Application of the KNN algorithm and the autoencoder neural network allowed us to fulfil this task with a very good accuracy. Thus, we displayed the utility of AI-based methodology in the research of ion channel gating, even in cases when the analyzed single-channel currents have apparently very similar characteristics.

Understanding the mechanism of channel activation and a proper description of spontaneous switching between stable conformations at versatile external conditions will allow the development of effective modulators of this group of transport proteins. The design of specific mitochondrial channel BK regulators acting only on the mitoBK channels within single cell type would be a milestone, since the majority of mitochondrial $K^{+}$ channel modulators exhibit a broad spectrum of off-target effects (uncoupling properties, inhibition of the respiratory chain, alteration of cellular Ca${}^{2+}$ homeostasis) [13,16]. Development of highly specific active substances demands not only a rigorous structural analysis of mitoBK α-β or α-γ complexes, but also indication of the functional and mechanical details of their activity. Unraveling the details of the whole molecular machinery of gating can be out of reach. Nevertheless, development of methods of classification and separation of the activity patterns representing different groups of channel isoforms with various types of auxiliary subunits can be a big step forward.

The input data for our analysis are obtained by the patch-clamp method, which is the most popular technique in investigation of ion channel dynamics [31]. It allows the obtaining of the time series of ionic currents flowing through a single channel at fixed external conditions (e.g., membrane potential $U_{m}$, concentration of channel-activating substances in pipette and bath solutions) in real time. The standard methods of kinetic analysis applied to the experimental patch-clamp results (i.e., probability of conducting states ($p_{op}$), activation curve ($p_{op}$ vs. $U_{m}$) etc.) can indicate some differences in channel dynamics stemming from the functional heterogeneity of the analyzed populations of ion channels. The recognition of kinetic differences is important step in the research of mechanical diversity of different mitoBK channel variants, but still it is not sufficient to unravel the details of their functioning. Potentially, it is possible, notwithstanding difficult, to use the kinetic parameters as an unequivocal discrimination criterion between various expression systems of the mitoBK channels. Unfortunately, the kinetic-based approach demands many repetitions of experiments and making additional assumptions about the separation criteria between the substates of the channel. We are convinced that machine learning can provide a complementary paradigm to the standard kinetic-based approach to ion channel physiology. An insightful analysis of the inherent features of the system dynamics within raw experimental data performed using AI algorithms ought to indicate and separate the individual, tissue-specific patterns of mitoBK channel activity.

In further applications, the existing AI-based software should be developed and supplied by tools allowing for correlation of signal characteristics with functional and structural features of a channel protein. The ability of modern machine-learning techniques to classify, identify, or interpret massive data sets implies their suitability to provide researchers a promising tool to unravel the details of gating machinery, including mechanistic discrepancies between different channel isoforms or types of α-β or α-γ assemblies in future investigations.

In this work we used the wild-type cells which may cause some minor problems in formulation of straight interpretation of the obtained results. Undoubtedly, our results showed that a sole way of how the single channel fluctuates within a relatively short time period (1000 data points which corresponds to 0.1 ms) can be considered to be a “fingerprint”. This short excerpt from the single-channel recording allows us to identify the cell type, where the investigated channel is located. The AI-based approach yields a good prediction accuracy even in cases when the channels exhibit a very similar level of open state probability (like at $U_{m}$ = +40 mV and $U_{m}$ = +40 mV; Figure 1, Table 1). Due to the use of the complex non-modified microbiosystems (wild-type cells) in our experiments, we are not able to indicate a single factor that diversifies the channel gating within the analyzed groups. The most striking difference between the patches of mitochondrial membrane from various cell types used in this study is that the investigated channels are co-assembled with different β subunits (2, 3 and 4), according to our previous results [32,33,34]. Thus, most of the inferences formulated here may pertain to the dynamical diversity of different mitoBK α-β complexes. Nevertheless, this rough simplification needs further investigations. To validate this view unquestionably, analysis of appropriately designed models should be performed i.e., gene-silencing models, post-translational modification of mitoBK, or co-expression of mitoBK channel with different β subunits in HEK or CHO cells.

We are convinced that it is highly interesting to carry out this kind of research on genetically modified systems. The literature suggests, specifically, that the β subunits exhibit tissue specificity in mitochondria [9]; however, their functional role for mitoBK physiology is not clearly reported. Our results show indirectly that they may modulate conformational dynamics of the mitoBK channels in such a detrimental way that the patch-clamp traces describing activities of different α-β complexes are distinguishable from each other.

In the studies summarized in [27] where the authors determined the long-awaited atomistic cryo-EM structures for the full-length human BK channel in complex with β4 subunit in high and low Ca${}^{2+}$ concentration regimes, it was noticed that identical gating conformations at high and low Ca${}^{2+}$ concentrations occur in the absence and presence of modulatory β4 subunit. In consequence, a conclusion emerged that the β4 ought to modulate the relative stabilities of ‘preexisting’ conformations rather than creating new ones during Ca${}^{2+}$-activation of the plasma-membrane BK channel. In general, the influence of β subunits on BK channel activation and gating in cell membrane, is relatively well-described in the literature [23,24]. In brief, the β2 and β3 are involved in BK channel inactivation. All of them can modulate the channel sensitivity to Ca${}^{2+}$ ions in an own specific way. Considering the mechanistic link between the channel α and β subunits, the studies suggest that the auxiliary subunits can interact with voltage-sensing domain, and affect the gating charge.

What is well worth mentioning, in this work we describe the differences in gating of mitochondrial BK channels, where the relation between the α-auxiliary subunits are not so broadly documented. In so far as the localization of the BK-DEC splice variant of BK-type channels is confirmed in mitochondria, it is still not known whether this isoform imposes functional changes in channel activity compared to the other BK isoforms present in cell membrane [9]. Moreover, plasma-membrane and inner mitochondrial membrane exhibit differences in biophysical properties resulting from various factors such as, e.g., the differences in lipid and protein composition, which can escalate the differences in channel gating in these two types of membranes. From this perspective, it is not certain whether there is a one-to-one correspondence between the possible interactions between α and auxiliary subunits (and its consequences on gating) in BK channels from plasma-membrane and their mitochondrial counterparts. Therefore, this aspect needs however further investigations with the use of the models including transfected HEK or CHO cells.

Beside the main focus of the current research, we showed the possible application of autoencoder neural networks to indicate anomalies within the analyzed data sets. Therefore, we showed the utility of the autoencoder in laboratory practice. In most studies, the desired results should describe a homogeneous population of the investigated systems (here, ion channels) without side-effects burden (some sudden changes in experimental conditions, inter-protein interactions, etc.). The applied procedure of anomaly detection allowed to point out experimental traces where the channel exhibited an unexpected kinetic state (notably higher or lower $p_{op}$ at given voltage than most channel proteins in the examined population), or some quite “good-looking” traces where the cascade of open-closed fluctuations exhibited anomalous switching. This kind of channel behavior can represent different modes of reactivity which can be caused by internal or external factors that are hard to identify and prevent). According to the obtained results, the efficiency of data classification was deeply affected by application of the anomaly detection technique in data processing. Specifically, the introduction of this stage of analysis allowed to significantly increase prediction accuracy (Table 1).

## 4. Methods and Materials

This section starts with the characterization of experimental details, i.e., preparation of material and data recording process. In the second part of the section, the methodology of data prepossessing and the set of implemented algorithms are described comprehensively.

### 4.1. Electrophysiology

The mitoBK channels investigated in this work were isolated from three independent cell types. The choice of cell lines was dictated by the dominating type of the mitoBK channel-β subunit complex. We aim to ensure versatility in this aspect. Thus, to analyze the mitochondrial BK assembled with β2 the commercially available human endothelial cell line (EA.hy926) was chosen [33] (derived from a human umbilical vein [44]). The mitoBK-β3 complex was represented in primary human dermal fibroblasts cell line (HDFa) [34] (commercial cell line). The mitochondrial BK in complex with β4 was investigated in the patches from embryonic rat mitochondria of the hippocampal neurons from pregnant female Wistar rats [32]. The whole procedure of hippocampal cells preparation as well as obtaining consequent mitochondrial pellets for further analysis are described in detail in [32].

As one can see, we decided to use one animal model to analyze features of mitoBK-β complexes. This decision was dictated by sufficiently good genetic alignment between rat and human BK channels (the percentage of the human sequence matching the sequence of rat orthologue is 98.30 %, as indicated by Ensembl release 100 [45]) from the point of view of our analysis, and high accessibility of the material.

The other cell cultures were performed as described in [33,34]. Specifically, the endothelial and fibroblast cells were grown in DMEM supplemented with 10% fetal bovine serum at 37 ${}^{\circ }$C in a humidified atmosphere with 5% CO${}_{2}$. The cell culture solutions contained also: 1% L-glutamine, 2% hypoxanthine hypoxanthine-aminopterin-thymidine, and 1% penicillin-streptomycin in case of endothelium, 2 mM L-glutamine, 100 U/mL penicillin, and 100 mg/ml streptomycin in case of dermal fibroblasts. All cells were fed and reseeded every third–fourth day.

#### 4.1.1. Mitochondria and Mitoplast Preparation

Differential centrifugation and hypotonic swelling were carried out to prepare fresh mitochondria and and subsequent mitoplasts, respectively, as described in [32,33,34]. Mitoplasts were prepared from the embryonic rat hippocampal neurons, the human endothelium and fibroblast mitochondria by incubation in a hypotonic solution (5 mM HEPES, 100 μM CaCl${}_{2}$, pH 7.2) for approximately 1 min, and then a hypertonic solution (750 mM KCl, 30 mM HEPES, and 100 μM CaCl${}_{2}$, pH 7.2) was subsequently added to restore the isotonicity of the medium. A fresh mitoplast was used for each repeating patch-clamp experiment.

#### 4.1.2. Patch-Clamp Experiments

The patch-clamp measurements were carried out in mitoplast-attached single-channel inside-out mode. The borosilicate patch pipettes (Harvard, UK) were pulled using a Flaming/Brown puller and reached a resistance of 10–20 MΩ. The isotonic solution filling the patch-clamp pipette contained: 150 mM KCl, 100 μM CaCl${}_{2}$ (endothelium, hippocampus) or 200 μM CaCl${}_{2}$ (fibroblasts), and 10 mM HEPES (fibroblasts, endothelium) or 5 mM HEPES (hippocampus) at pH 7.2. The composition of the pipette solution ought to ensure the full Ca${}^{2+}$-activation of the mitoBK channels under investigation. All patch-clamp experiments were carried out in an air-conditioned room at the temperature 24 ${}^{\circ }$C. The patch-clamp amplifier Axopatch 200 B was used in all recordings. The experimental signals were low-pass filtered at a corner frequency of 1 kHz and the sampling rate was given by the frequency of 4.00 kHz (at time intervals of 250.00 μs) set by the Clampex software. Single-channel currents were obtained in a voltage clamp mode at pipette potentials fixed at: −60, −40, −20, 20, 40, and 60 mV. The measurement error of single-channel currents was ΔI = 1 × 10^−6^ pA (implied by the equipment). Each experimental time series comprised N = 2 × 10^5^ current values. For each cell type we used 6–11 independent patches to record time series of single mitoBK channel currents at each value of membrane potential. In the case of endothelium we recognized some untypical levels of channel activity, i.e., “lowered” one, which probably stem from the inactivation of the mitoBK channel conferred by the interactions with β2 subunits [46,47]. These traces of untypical channel activity were not taken to further analysis. As a result a minimal number of independent recordings representing each combination of experimental conditions after were 3 samples (at each level of membrane potential and for each cell type).

#### 4.1.3. Basic Analysis of Experimental Results

For each experimental recording we carried out a basic analysis, where the open state probability ($p_{op}$) was evaluated. In this aim we followed the procedure previously described in [48]. In brief, the most prominent stage to calculate $p_{op}$ was to find the threshold current value (TC), which separates the conducting and non-conducting states. In that aim first we plot the probability density function of recorded currents approximated by the nonparametric kernel density estimate with Epanechnikov kernel in log-log scale. Then, the intervals are found where power law is satisfied. The last stage is to find the point intersection of the power law plots; this point directly indicates the TC.

### 4.2. Machine-Learning Approaches

**Figure 4:**
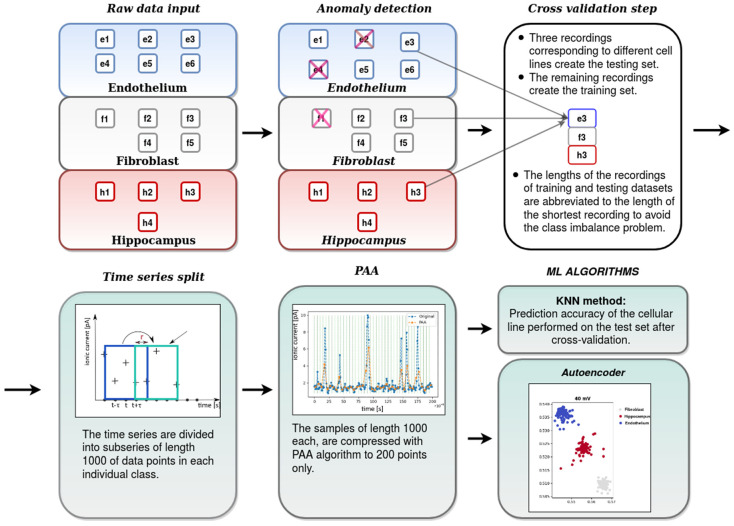
The scheme presenting the subsequent stages of the patch-clamp data processing.

The subsequent stages of our analysis are summarized in Figure 4.

Before feeding the data into the machine-learning algorithm it is necessary to properly preprocess it. The preprocessing stages employed in our approach are depicted in the Figure 4 and explained in detail in the undermentioned subsection.

#### 4.2.1. Data Preprocessing

The raw experimental data coming from the patch-clamp experiment consists of several recordings (long ion channel currents) for each cell type and each membrane potential $U_{m}$. At the initial processing stage, we apply the anomaly detection technique using the autoencoder neural network and reject the recordings which clearly stand out from the rest. You can find more detailed explanation of the adopted procedure in Section 4.2.3.

**Figure 5:**
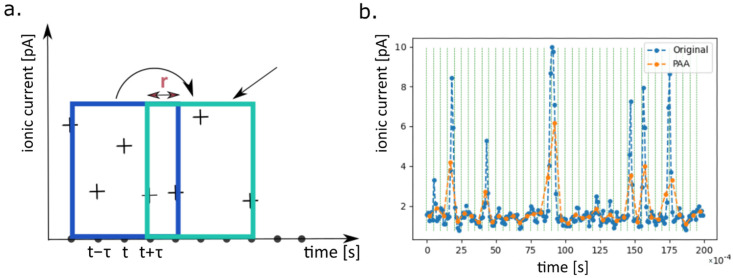
(**a**) Illustration of the time series split. In the current analysis the r=200. (**b**) A schematic representation of the PAA method. Each original subseries of 1000 data points (which corresponds to the time interval of 0.1 s at the applied sampling rate of 10 kHz) is “compressed” to the series of 200 points by the PAA algorithm of dimensionality reduction. Here, for clarity we presented the dimensionality reduction performed on the original subseries of 200 points (corresponding to the time interval 0.02 s). As one can see, application of PAA algorithm allow the retention of the main features of the original input series.

Afterwards, we split the remaining recordings into the training and testing datasets. We adopt the following train-test split strategy: in each cross-validation step we choose one recording of each cell type. These three recordings create the testing dataset, while the remaining ones are treated as the training dataset. Then, in order to avoid the “class imbalance problem” [49], with several recordings of different lengths in each of the datasets, we truncate them to the length of the shortest recording. In the next step, in order to prepare the data for further calculations, we split the recordings into the smaller time series, each of length 1000 data points (which corresponds to the time interval of 0.1 ms as the applied sampling rate was equal to 10 kHz). The stride *r* of the created subseries is equal to 200, i.e., one takes first 1000 points from the original subseries, then points from 201 to 1200, afterwards from 401 to 1400 and so on. This idea is presented on the left-hand side of the Figure 5. In this manner, from ever recording of the length *L*, (L−l)/r samples of length *l* are obtained (in the case that the result is not an integer it is rounded down).

From the above-mentioned procedure one obtains the samples, each of the length 1000. The length of each sample in the datasets is then reduced by the Piecewise Aggregate Approximation (PAA) [50]. Using this method, the length of sample is reduced from 1000 to 200 points. Importantly, despite this reduction, the characteristic features of the record are conserved (see Figure 5). In the end, all data points are normalized into the range [0,1].

Afterwards, we feed the preprocessed training data into the ML algorithm, calculate its performance and change the data division: one of the recordings which was previously put into training dataset is now in test data and vice-versa. We repeat all procedure concerning the preprocessing part and once again calculate the performance of our model. We then iterate over all possible configurations of the training-testing datasets. In the end, the overall model performance is averaged over all such cross-validation steps.

#### 4.2.2. Autoencoder

An autoencoder is a neural network that is trained to attempt to copy its input to its output. In other words, it aims at finding the non-linear identical function f which maps the input data X in itself [51]. Since the data labeling in this method is not required, the autoencoder, in contrast to the other neural networks, is considered to be the unsupervised machine-learning technique [52]. As well as the Principal Component Analysis algorithm (PCA), it can be used for the dimensionality reduction. However, due to its non-linearity it is more accurate and can capture more non-trivial features of the data [53]. In its simplest form the autoencoder consists of two parts: an encoder and a decoder. The encoder E transforms the input data *X* into the latent space *L*, which usually has a lower dimensionality than the input space *X*. The decoder D transforms back the obtained latent space *L* into the reconstructed one $X^{\prime }$:

(1) $$E\left(X\right)=L,\;D\left(L\right)=X^{\prime }.$$

**Figure 6:**
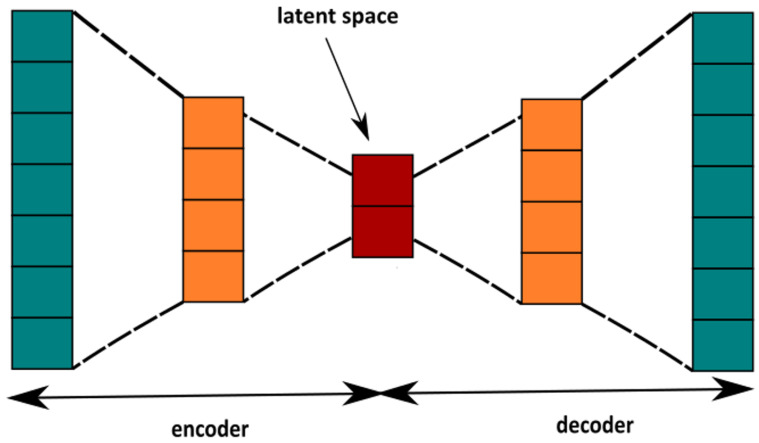
The scheme of autoencoder. The green squares stand for the input data, the orange ones indicate the intermediate layer of neurons. The red squares denote the latent space and provide the reduced representation of the sample.

This general idea of the autoencoder is presented in the Figure 6.

The autoencoder typically has the form of the feed-forward neural network consisting of several layers. The main task of the neural networks E,E is to make the reconstructed data $x^{\prime }$ as close as possible to the original input *x*. It is realized by minimizing the average reconstruction error J:

(2) $$J=\frac{1}{N}\sum_{i=1}^{N}\left|\right|x_{i}^{\prime }-x_{i}{\left|\right|}^{2},$$

where *N* is the number of samples in the training set *X* and $x_{i},\;x_{i}^{\prime }$ stand for the input and output sample, respectively.

The encoder part of our autoencoder, consists of the 200-dimensional input layer, 2 intermediate layers containing 100 and 50 neurons, and the 2-dimensional bottleneck layer at the end. As the activation function we incorporated the sigmoid function σ(x):

(3) $$\sigma \left(x\right)=\frac{1}{1+e^{-x}}.$$

The architecture of the decoder is reversed in comparison to the encoder.

#### 4.2.3. Anomaly Detection with Autoencoder

**Figure 7:**
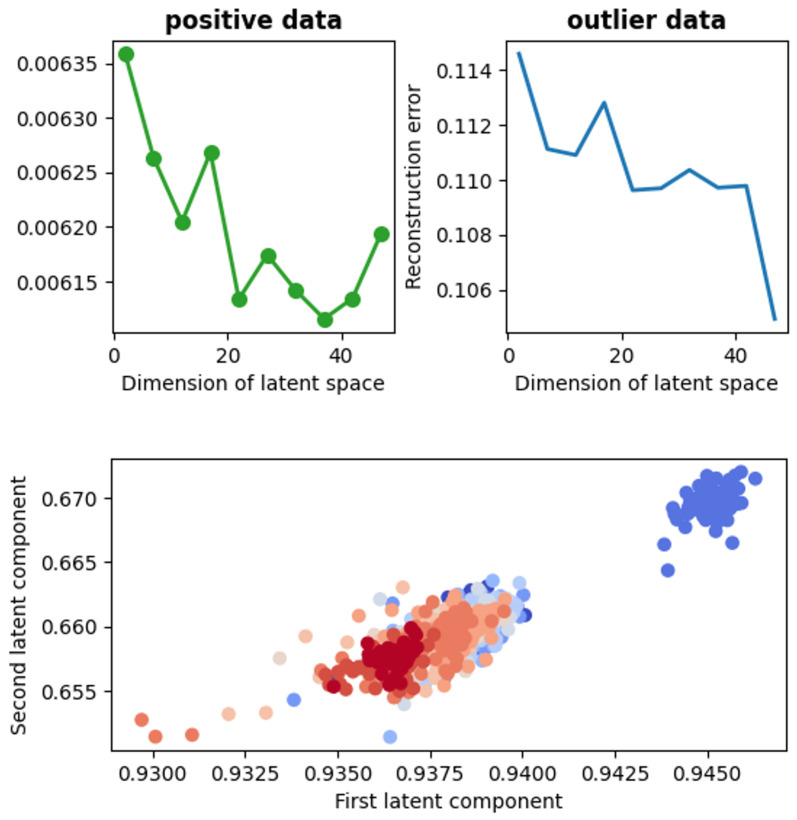
The representation of the applied technique of anomaly detection. The plot at the bottom shows the representation of the data corresponding to the fibroblast cell line at 40 V in 2-dimensional space after its compression with the autoencoder. Different colors in the figure correspond to the independent patches of the same cell line. Each dot represents a 1000-data-points-long subseries from the original recording. As one can see, within the recordings represented by the dark blue dots and the orange ones there were some intervals where the activity of the channel stands out from its typical characteristics represented by the cluster at the top (dark blue) and some orange dots at the left bottom. These outstanding points are called here “outliers”. The plots on the top illustrate the reconstruction error dependence on the dimension of the latent space for positive (typical) and outlier data, respectively.

To deal with the problem of anomalies in our data, we apply the autoencoder, which serves as a great tool for unsupervised outlier removal [54]. First, we prepare the recordings corresponding to different categories (cell type, voltage) and handle it according to the procedure described in the above subsection. Then, we treat such preprocessed data as our training set and try to reconstruct it with the autoencoder. To make the visualization of the compressed data possible, we assume the 2-dimensional latent space. Exemplary results are presented on the left side of the Figure 7. Various colors correspond to different recordings representing data belonging to the same category.

In the Figure 7 it is clearly visible that one record stands out. From the technical point of view the patch-clamp method is very sensitive to internal and external factors. Since autoencoder is a method specialized in finding anomalies in time series, this technique was also used here for this purpose. Thus, this kind of outstanding behavior can stem from e.g., a protein-protein interaction performed by the channel with another membrane protein. To avoid the underestimation of obtained results we decide to remove the experimental traces assigned as anomaly from the further analysis. To make sure that the record is really an outlier, we choose, based on the visualization, one positive and one anomaly record. Then, for both kinds of data, one calculates the reconstruction error J in function of the bottleneck dimension. The result of this calculation is shown on the right-hand side of the Figure 7.

As stated in the paper [54], the outlier and positive data can be discriminated by the magnitude of the reconstruction error J. It happens also in our case (see the right-hand side of the Figure 7).

At the end, our data is fed into the K-nearest neighbors classifier (KNN) as described in the section below.

#### 4.2.4. PAA

The PAA (Piecewise Aggregate Approximation) of time series is an extremely simple method allowing for the down-sampling of the original time series. In short, in order to reduce the dimensionality of the time series of length *l* to the length *w* (*w* < *l*) we need to divide it into equal *w*-dimensional “frames” and calculate the mean value of each “frame”. The new, reduced representation of the initial time series consists of these average values. An exemplary application of the PAA method is presented on the right-hand side of the Figure 5.

#### 4.2.5. K-Nearest Neighbors Algorithm

The K-Nearest Neighbors Classifier (KNN) is an example of supervised algorithms. In our work we used this algorithm is supported by the results of the autoencoder neural network, but this method also has some individual successes in the field of time series analysis [35,55]. The intuition behind the KNN algorithm is one of the simplest of all supervised machine-learning algorithms. In this technique the distance between new data points to all other training data points is calculated. Then the selection of K-nearest neighbors is carried out, to finally assign a data point to the class in which the most K data points belong.

**Figure 8:**
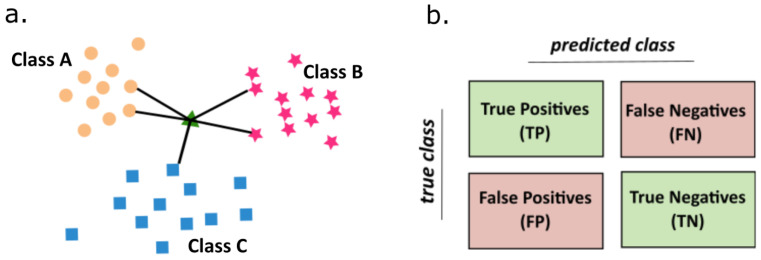
(**a**) Representation of K-nearest neighbors algorithm: general scheme of algorithm, (**b**) illustration of confusion matrix.

Therefore, the main idea of the KNN algorithm is that a given object can be classified to the class which is most frequently represented among its K-nearest neighbors from the training set. It is presented in Figure 8.

The essence of the KNN methodology algorithm can be summarized in the following points:

1. The values of the parameter K and the distance metric *d* are selected. This prior selection has a great impact on the obtained results. The values of K is a key choice that gives a balance between overtraining and the under-fitting. In our case K = 5 was the optimal selection. As a metric of distance a simple Euclidean relation was used (4).
  (4) $$d\left(x_{i},x_{j}\right)=\sqrt{\sum_{i=1}^{N}{\left(x_{i}-x_{j}\right)}^{2}},$$
  where *N* is a space dimensionality.
2. For a sample subjected to the classification the number of K-nearest neighbors are determined by the calculation of distance to the nearest neighbors. At the final step, a class etiquette is assigned by majority of vote. Thus, the KNN algorithm classifies the object to this class, which is most frequently represented among K-nearest neighbors from the training set.
3. To avoid the problems with the overfitting or the selection bias [56] the cross-validation technique is applied. One step in this technique is about partitioning the data into training and test sets. In our case, in every round, one record from each cellular line, serves as a test set, whereas the remaining data forms the training set. We make so many rounds, each involving a different collection of the testing records.

The prediction accuracy is a performance measure that is calculated based on the overall confusion matrix, obtained after summing the number of correctly classified samples in the test set coming from all cross-validation steps. The confusion matrix is a kind of table layout allowing the visualization of the model performance. An exemplary confusion matrix is shown in Figure 8b. In that example one has two classes known as ’positive’ and ’negative’. True Positives (TP) represent the samples which were ’positives’ and were classified as such. Similarly, True Negatives (TN) are correctly classified ’negatives’. The remaining two categories denote the misclassified samples. The extension of the confusion matrix to the cases with more classes is straightforward. In each case, no matter the number of classes, it is defined as the ratio of the number of samples lying on the diagonal to the number of all samples. Thus, in the case of the binary confusion matrix presented in Figure 8b the accuracy would be computed as:

(5) $$Acc\;=\;\frac{TP+TN}{TP+TN+FN+FP}\cdot 100\%.$$

#### 4.2.6. K-Means

The K-means method of clustering is one of the most popular techniques of unsupervised machine learning. It was used for the first time in 1967 by Macqueen et al. [57]. The main aim of this method is to search and extract the groups of related objects, and arrange them into clusters. In simple words this technique identifies *K* number of clusters and assigns each subsequent data point to the nearest cluster. The method can be characterized as follows:

1. the number of clusters is determined. Their centroids are chosen randomly.
2. the kind of metric is selected. Similar to the KNN algorithm, the Euclidean distance is the most popular choice.
3. the distance from all points to centroids is determined. The new object is assigned to the cluster with the closest center.
4. based on the mean of points assigned to a given cluster, the new centers of concentration are set down.

Steps 3 and 4 are repeated until convergence.

## Figures and Tables

**Table 1:** Prediction accuracy of the cell lines performed on the test data set after cross-validation. First column indicates the pipette potential used in the experiment. Second and third columns show the accuracy without and with application of the anomaly detection technique, respectively. The remaining three columns present the average distances between the data corresponding to the different types of cells after its compression to the 2-dimensional latent space. The following abbreviations are introduced: Dist FH-the distance between the clusters representing fibroblasts and hippocampal cells; Dist FE-the distance between the clusters representing fibroblasts and endothelial cells, and Dist HE-the distance between the clusters representing hippocampal and endothelial cells.

Pipette Potential [mV]	Acc without AD	Acc with AD	Dist FH $\left[10^{-3}\right]$	Dist FE $\left[10^{-3}\right]$	Dist HE $\left[10^{-3}\right]$
−60	77%	100%	4.26	8.38	10.34
−40	59.9%	95.9%	5.68	9.29	10.61
−20	84.3%	84.3%	5.94	10.29	14.1
+20	75.5%	96.5%	2.52	2.37	4.13
+40	72.9%	99.46%	10.91	12.63	6.35
+60	96.6%	96.6%	3.73	7.15	9.18

## Data Availability

Experimental data was obtained by P.B. partially at Nencki Institute of Experimental Biology, Warsaw, Poland and Otto-von-Guericke-University, Magdeburg, Germany with the permission of Directors of these institutions. Thus, the datasets generated and analysed during the current study are available on request from the corresponding author, who will inform the representatives of the mentioned institutions about the data reuse.
